# The cardiac and haemostatic effects of dietary hempseed

**DOI:** 10.1186/1743-7075-7-32

**Published:** 2010-04-21

**Authors:** Delfin Rodriguez-Leyva, Grant N Pierce

**Affiliations:** 1 Department of Physiology, University of Manitoba and Institute of Cardiovascular Sciences, St Boniface Hospital Research Centre, 351 Tache Avenue, Winnipeg, Manitoba, R2H 2A6, Canada; 2 Cardiovascular Research Division, V.I. Lenin Universitary Hospital, s/n Lenin Avenue, Holguin, 80100, Cuba

## Abstract

Despite its use in our diet for hundreds of years, hempseed has surprisingly little research published on its physiological effects. This may have been in the past because the psychotropic properties wrongly attributed to hemp would complicate any conclusions obtained through its study. Hemp has a botanical relationship to drug/medicinal varieties of Cannabis. However, hempseed no longer contains psychotropic action and instead may provide significant health benefits. Hempseed has an excellent content of *omega-3 *and *omega-6 *fatty acids. These compounds have beneficial effects on our cardiovascular health. Recent studies, mostly in animals, have examined the effects of these fatty acids and dietary hempseed itself on platelet aggregation, ischemic heart disease and other aspects of our cardiovascular health. The purpose of this article is to review the latest developments in this rapidly emerging research field with a focus on the cardiac and vascular effects of dietary hempseed.

## Introduction

*Cannabis sativa L*. is an annual plant in the Cannabaceae family. It has been an important source of food, fiber, medicine and psychoactive/religious drug since prehistoric times [1]. *Cannabis *is mentioned as a medication in ancient Egyptian medical texts: Ramesseum III Papyrus (1700 B.C.), Eber's Papyrus (1600 B.C.), the Berlin Papyrus (1300 B.C.), and the Chester Beatty VI Papyrus (1300 B.C.) [1,2].

Two main types of Cannabis Sativa L. must be distinguished, the drug and non-drug types. The first is also known as marijuana, hashish or Cannabis tincture and contains Δ^9^-Tetrahydrocannabinol (THC) in concentrations between 1-20%, high enough to exhibit psychoactivity. The second type of *Cannabis Sativa L*. is industrial hemp with THC concentrations < 0.3% so it has no psychoactive properties [3,4].

Canada, Australia, Austria, China, Great Britain, France and Spain are among the most important agricultural producers of hempseed. In the United States, it is not legal to cultivate hempseed. This is primarily because many believe that by legalizing hemp they may lead to a legalization of marijuana [5]. Other governments have accepted the distinction between the two types of Cannabis and, while continuing to penalize the growing of marijuana, have legalized the growing of industrial hemp [5].

Hempseed possesses excellent nutritional value. It is very rich in essential fatty acids (EFAs) and other polyunsaturated fatty acids (PUFAs). It has almost as much protein as soybean and is also rich in Vitamin E and minerals such as phosphorus, potassium, sodium, magnesium, sulfur, calcium, iron, and zinc [6,7]. The nutrient profile of hempseed is shown in Table 1. Hempseed oil contains all of the essential amino acids and also contains surprisingly high levels of the amino acid arginine, a metabolic precursor for the production of nitric oxide (NO), a molecule now recognized as a pivotal signaling messenger in the cardiovascular system that participates in the control of hemostasis, fibrinolysis, platelet and leukocyte interactions with the arterial wall, regulation of vascular tone, proliferation of vascular smooth muscle cells, and homeostasis of blood pressure [8]. In a study that included 13 401 participants, 25 years and older from the Third National Health Nutrition and Examination Survey, an independent relationship was shown between the dietary intake of L-arginine and levels of C-Reactive protein [9], a marker strongly correlated with the risk of cardiovascular disease (CVD) [10]. The results of this populated-based study suggested that individuals may be able to decrease their risk for CVD by following a diet that is high in arginine-rich foods [9]. Dietary hempseed is also particularly rich in the omega-6 fatty acid linoleic acid (LA) and also contains elevated concentrations of the omega-3 fatty acid α-linolenic acid (ALA). The LA:ALA ratio normally exists in hempseed at between 2:1 and 3:1 levels. This proportion has been proposed to be ideal for a healthy diet [11]. Other rich sources of LA [12,13] are listed in Table 2.

**Table 1:** Nutrient profile of hempseed*.

Nutrient	Units	Value per 100 grams	Nutrient	Units	Value per 100 grams
**Proximates**					

Energy	kcal	567	**Lipids**		

Energy	kJ	2200	Saturated fat	g	3.3

Protein	g	24.8	16:0	g	3.44

Total lipid (fat)	g	35.5	18:0	g	1.46

Ash	g	5.6	20:0	g	0.28

Carbohydrates	g	27.6	Monounsaturated fat	g	5.8

Fiber, total dietary	g	27.6	18:1n9	g	9

Digestable fiber	g	5.4	Total polyunsaturated	g	36.2

Non-digestable fiber	g	22.2	18:2n6	g	56

Moisture	g	6.5	18:3n6	g	4

Glucose	g	0.30	18:3n3	g	22

Fructose	g	0.45	18:4n3	g	2

Lactose	g	<0.1	Cholesterol	mg	0

Maltose	g	<0.1	**Amino acids**		

			Tryptophan	g	0.20

**Minerals**			Threonine	g	0.88

Calcium, Ca	mg	145	Isoleucine	g	0.98

Iron, Fe	mg	14	Leucine	g	1.72

Magnesium, Mg	mg	483	Lysine	g	1.03

Phosphorus, P	mg	1160	Methionine	g	0.58

Potassium, K	mg	859	Cystine	g	0.41

Sodium, Na	mg	12	Phenylalanine	g	1.17

Zinc, Zn	mg	7	Tyrosine	g	0.82

Copper, Cu	mg	2	Valine	g	1.28

Manganese, Mn	mg	7	Arginine	g	3.10

Selenium, Se	mcg	<0.02	Histidine	g	0.71

**Vitamins**			Alanine	g	1.28

Vitamin C	mg	1.0	Aspartic acid	g	2.78

Thiamin	mg	0.4	Glutamic acid	g	4.57

Riboflavin	mg	0.11	Glycine	g	1.14

Niacin	mg	2.8	Proline	g	1.15

Vitamin B-6	mg	0.12	Serine	g	1.27

Vitamin A	IU	3800			

Vitamin D	UI	2277.5			

Vitamin E	mg	90.00			

* Adapted from reference 6 and 7. Data based on Finola variety of hempseed.

**Table 2:** Rich sources of the essential fatty acid linoleic acid*.

Source of LA	LA (g/100 g)	ALA (g/100 g)	Ratio n6/n3
Safflower oil	73	0.4	>100
Corn oil	57	1	57
Hempseed Oil	56	22	2.5
Cottonseed oil	50	0.2	>100
Soybean oil	50	8	6.2
Sesame oil	40	0.3	>100
Black walnuts	37	2	18.5
English walnuts	35	6.8	5.1
Sunflower seeds	30	0.06	>100
Brazil nuts	25	0.01	>100
Margarine	22	2.1	10.4
Pumpkin and squash seeds	20	0.12	>100
Spanish peanuts	16	0.01	>100
Peanut butter	15	0.08	>100
Almonds	10	0.06	>100

*Adapted from reference [12] and [13]

**Figure 1:**
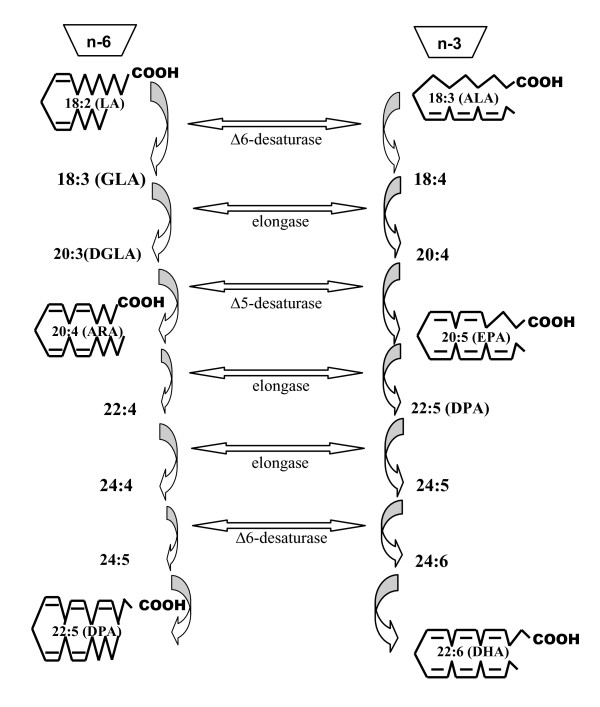
**Biochemical pathway for linolenic acid and α-linolenic acid transformation**. ALA = α-linolenic acid; ARA = arachidonic acid; DGLA = dihomo γ-linolenic acid; DHA = docosahexaenoic acid; DPA = docosapentaenoic acid; EPA = eicosapentaenoic acid; GLA = γ-linolenic acid; LA = linoleic acid.

The long chain PUFA that is found in the body ultimately originates from the diet and through elongation and desaturation of their dietary precursors, ALA and LA. Both families of fatty acids, n-3 and n-6, share and compete for the same enzymes (Δ^6^-desaturase, Δ^5^-desaturase, and elongases) in their biosynthetic pathways. The Δ^6^-desaturase enzyme is the rate-limiting step [9]. Following its metabolism, LA can be converted into arachidonic acid whereas ALA will be converted into the long chain fatty acids, eicosapentaenoic acid (EPA) and docosahexaenoic acid (DHA) (Figure 1). A high LA intake interferes with the desaturation and elongation of ALA [14]. Therefore, theorically, a lower ratio of omega-6/omega-3 fatty acids is more advantageous in reducing the risk of many of the chronic diseases of high prevalence in Western societies. The ratio of ω-6 to ω-3 fatty acids ranges from 20-30:1 in Western societies instead of the traditional (historic) range of 1-2:1 on which human beings evolved [15]. This is thought to be closely associated with chronic diseases like coronary artery disease, hypertension, diabetes, arthritis, osteoporosis, inflammatory and autoimmune disorders and cancer.

Hempseed is also a rich and unusual source of the polyunsaturated fatty acid gamma linolenic acid (GLA) (18:3n6) to the body. Additionally, another important biological metabolite of ALA and LA, stearidonic acid (18:4n3; SDA) is also present in hempseed oil (Figure 1). Both can inhibit inflammatory responses [16,17].

Recently, many studies have demonstrated that dietary interventions can play a central role in the primary and secondary prevention of several diseases. The PUFAs derived from fish, EPA and DHA, have been extensively studied. Based on the close relation between the pathways that metabolize ALA and LA, and the capacity of both to be converted into long chain fatty acids, plant sources of ALA (i.e. flaxseed, canola and others) have begun to attract more scientific attention for their potential to improve our health. However, because of legal regulations, lack of knowledge and some confusion about the differences between fiber hemp and marijuana, the growth of hempseed research has been slower than expected. In view of its long history of dietary usage, it is surprising that research on the effects of dietary hempseed in animal and humans has been limited. Furthermore, because of its expected nutritional value and the hypothetical benefits of LA and ALA against a variety of health disorders, a better understanding of the appropriate doses and presentation (oil, seed, etc) of hempseed should represent useful health-related information. It is important to point out that dietary hempseed as an energy containing food item introduces changes in the fatty acid composition of the diet and will inevitably replace other dietary components under an isocaloric condition. Previously some [18] but not all authors [19] have found differences in body weight after the administration of 30 ml/d of hempseed oil for four to eight weeks in humans. Finally, an identification of the target patient population (age, clinical condition, co-morbidities, etc) that may benefit the most from a supplementation of hempseed in the diet would also be important information.

## Animal Data

The biochemical metabolism of omega-6 fatty acids like LA produces eicosanoids in the body. Eicosanoids are biologically active and contribute to the formation of thrombi and atheromas and shifts the physiological state to one that is prothrombotic and proaggregatory, with increases in blood viscosity, vasospasm, and vasocontriction and decreases in bleeding time [15]. Hempseed is rich in LA content. Therefore, hempseed has received research attention for its effects on platelet aggregation.

Richard et al [20] reported that diets supplemented with 5% and 10% hempseed (wt/wt) for 12 weeks resulted in a significant increase in total plasma PUFAs in rats. ALA and LA levels increased significantly in a concentration-dependent manner [20]. Dietary hempseed supplementation also resulted in a significant inhibition of platelet aggregation and a lower rate of aggregation. This is an important result with physiological and pathological implications. As we have become increasingly aware of the importance of blood clots to the initiation of myocardial infarctions and strokes, the capacity of a dietary intervention like hempseed to inhibit clot formation has obvious appeal. However, if excessive bleeding is an expected event (as would be the case during surgery), it becomes essential for the physician/surgeon to know of a prior history of dietary hempseed usage.

These data on the effects of dietary hempseed on platelet aggregation have been extended into hypercholesterolemic conditions by Prociuk and colleagues [21]. They have shown that rabbits fed a high cholesterol diet for eight weeks exhibit an enhanced platelet aggregation [21]. However, when 10% hempseed was supplemented to the diet together with the high cholesterol diet, these hypercholesterolemic animals displayed normal platelet aggregation values. This normalization was not related to any correction of the elevated plasma cholesterol levels but was related in part to the increased levels of plasma gamma-linolenic acid [21]. Because most patients at high risk for coronary heart disease are hypercholesterolemic, these findings have important potential for treating or preventing cardiovascular diseases.

Two other studies have been focused on the capacity of hempseed for altering cardiac function before and after an ischemic event [22,23]. Both studies have shown no effects of a hempseed-supplemented diet on basal cardiac contractile function or electrical activity before ischemia [22,23]. However, the data on the effects of dietary hempseed on cardiac performance post-ischemia is less consistent. Al-Khalifa and colleagues [23] reported that hearts from rats fed a 5% or 10% hempseed supplemented diet for 12 weeks exhibited significantly better post-ischemic recovery of maximal contractile function and enhanced rates of tension development and relaxation during reperfusion than hearts from the control group. The authors found that these hearts were not protected from the occurrence of premature contractions, nor were the increases in resting tension altered during ischemia or reperfusion [23]. This beneficial effect of hempseed on post-ischemic cardiac performance may be species specific. The same lab found that supplementation of the diet with 10% hempseed in rabbits did not show any beneficial effects on left ventricular end-diastolic pressure (LVEDP), left ventricular developed pressure (LVDP), arrhythmia incidence and arrhythmia duration during ischemia and reperfusion [22]. Some limitations of the study related to the duration of the dietary intervention (8 weeks as opposed to 12 weeks) and sample size may have influenced the capacity for the dietary hempseed to protect the heart during ischemic insult [22].

## Clinical Data

The actions of dietary hempseed in humans have only been studied to a limited extent. Fatty acid bioavailability from hempseed oil was recently studied in comparison to two other dietary oils (fish and flaxseed) [24]. Hempseed and hempseed oil is enriched in LA and GLA. Eighty-six healthy subjects completed a 12 week dietary supplementation with 2 g/day of these oils. The hempseed intervention did not significantly increase the concentration of LA, GLA or any other fatty acid in the plasma of the subjects, nor did it change the level of plasma total cholesterol (TC), high density cholesterol (HDL-C), low density cholesterol (LDL-C) or triglycerides (TG) [24]. Both flaxseed and fish oils did induce significant changes in circulating fatty acid species associated with their respective oils (ALA for flaxseed; EPA and DHA for fish oil) [24]. Supplementation with hempseed oil also did not induce any change in collagen- or thrombin-stimulated platelet aggregation or in the levels of circulating inflammatory markers [24]. It was suggested that the lack of effects may be related to the dose used [24]. This hypothesis has been supported by data obtained in another dietary intervention that used higher doses of hempseed (30 ml/day) [18]. In this randomized, double-blinded, crossover design trial, hempseed and flaxseed oils were compared at the same doses. After 4 weeks of supplementation, the hempseed intervention increased the concentrations of both LA and GLA in serum cholesteryl esters (CE) and TG. The flaxseed intervention resulted in higher serum CE and TG concentrations of ALA. However, a statistically significant decrease in GLA concentrations was observed during this period of intervention. Importantly, the proportion of arachidonic acid in CE was lower after the flaxseed diet than after the hempseed supplementation but this was not statistically significant. However, the hempseed supplements resulted in a lower total cholesterol:HDL cholesterol ratio. A higher total-to-HDL cholesterol ratio has been found in association with coronary heart disease [25]. However, no significant differences were found between the effects of flaxseed and hempseed oils in terms the fasting serum total or lipoprotein lipid levels, plasma glucose levels, or insulin or hemostatic factors [18]. Callaway and colleagues [19], using 30 ml/day of hempseed oil, conducted a 20-week randomized, single-blind crossover study in 20 patients with atopic dermatitis, and found that the levels of both essential fatty acids, LA and ALA, and GLA increased in all lipid fractions after using hempseed oil, with no significant increases of arachidonic acid in any lipid fractions. Moreover, atopic dermatitis symptoms were improved after the intervention with hempseed oil [19].

These results emphasize the importance of using higher doses of hempseed oil if significant increases in fatty acid species are to be achieved. Clearly, the ingestion of two large capsules of hempseed daily (as most people in the general public may ingest), is insufficient to achieve a desired increase in LA or GLA levels in the plasma [24]. Much larger doses are required to induce beneficial physiological effects. However, this may not be possible to achieve currently in the general population. If 10-15 times the amount used by Kaul and co-workers [24] is required to achieve a significant increase in plasma fatty acid levels, it would be unpractical to expect the general public to ingest 20-30 capsules of hempseed per day. This is a significant problem that the food and supplement industry must address in the future if hempseed is to be considered a realistic dietary approach to healthy living. Supplementing the diet with tablespoons of hemp oil in addition to hemp capsules as well as ingesting foods that contain these omega-3 fatty acids may be the optimal way to obtain them.

## Linoleic acid and heart disease: New research fields for hempseed

Hempseed is a rich source of LA and others nutrients. The specific pathologies or conditions in which it can be used effectively are in need of more research but the data presently available suggest that LA may have beneficial effects in certain cardiovascular circumstances.

### Effects on cholesterol levels

Iacono et al [26] reported that a high LA based diet (10.8%) decreased total cholesterol by 15% and LDL-C by 22%, without producing significant changes in plasma HDL-C after 6 weeks of dietary intervention in 11 healthy middle aged, male subjects. Apolipoprotein B decreased by 37% whereas apolipoprotein A-I increased by 24% in the group of individuals supplemented with this diet [26]. In a multiple crossover design that included 56 normolipemic, healthy subjects, Zock and colleagues [27] found that those who received the LA supplemented dietary intervention for three weeks (2.0% of total energy intake as LA) obtained lower levels of serum LDL-C, and higher HDL-C levels when compared with subjects who received its hydrogenation products elaidic (trans-Cl8:ln9) and stearic acid (C18:O). Recently, Mensink et al [28] employed a meta-analysis that included 60 controlled trials to show that polyunsaturated fat (mainly LA) reduces LDL-C, triglycerides and increases HDL-C. However, others have shown that healthy individuals supplemented for 4 weeks with hempseed exhibited a lower total-to-HDL cholesterol ratio [24]. A higher total:HDL cholesterol ratio is associated with coronary heart disease and has a worse prognosis after a myocardial infarction [29,30]. Clearly, the issue is not resolved yet. The population studied (healthy vs clinically compromised), the dosages of hempseed used, the presentation administered (whole hempseed vs milled hempseed vs hemp oil vs purified LA), the duration of the dietary intervention, the composition of the diet, are all factors that may be critical in producing the effects (of lack of effects) in these studies. More research is needed in order to understand if these specific conditions influence cardiovascular efficacy and to understand which metabolic factors are most sensitive (hypertriglyceridemia, hypercholesterolemia, low HDL-C, or other hyperlipoproteinemias) to this kind of dietary intervention.

### Effects on high blood pressure

Results reported by The International Study of Macro-Micronutrients and Blood Pressure, a cross-sectional epidemiological study that included 4680 individuals, suggested that dietary LA intake may contribute to prevention and control of high blood pressure [31]. Other small studies have found that supplementation with LA (4 g-23 g/day) decreased blood pressure after 4 weeks of dietary intervention [32,33]. However, these promising results are in conflict with another study that reported no association between LA intake and lower blood pressure levels [34]. Studies using hempseed as a source of LA for hypertensive patients have not been conducted. It is also important to note that the consequences of these kinds of diets on arterial stiffness and vascular perfusion characteristics are unknown. The additional effects of these diets on ventricular hypertrophy that develops secondary to high blood pressure is not known nor are the effects when hempseed is supplemented with an antihypertensive medication. The potential for hempseed to alter drug kinetics in the body has not been studied.

### Effects on atherosclerosis

Almost three decades ago, Cornwell and Panganamala postulated that an intracellular deficiency in essential fatty acids plays a central role in the atherogenic process [35]. Recently, Das [36] showed how a defect in the activity of Δ^6 ^and Δ^5 ^desaturases may be a factor in the initiation and progression of atherosclerosis. He also provided evidence that low-grade systemic inflammatory conditions are also essential fatty acids deficient states [36]. With our current understanding of the close relationship that infectious disease and inflammation has with atherogenesis [37,38], it is not difficult to predict that foods with an optimal LA-ALA ratio will reduce inflammation under ideal dietary conditions and it may thereby attenuate atherosclerotic heart disease. Unfortunately, the effects of LA on atherosclerosis are not completely clear. Arachidonic acid can be derived from LA. This can be converted to prothrombotic and proinflammatory prostaglandins. However, changes in dietary LA within the usual dietary range do not appreciably alter arachidonic acid levels [39,40]. Consistent with this, some have suggested that LA could have anti-inflammatory effects mediated by biochemical pathways that do not involve the cyclooxygenase pathway [41]. Presently, the randomized, controlled trials that address this topic have not been able to distinguish between the effects of omega-3 and omega-6 fatty acids [42]. Both have had beneficial effects by decreasing plasma levels of soluble TNF receptor 1 and 2, indicators of TNF activity [42].

Surprisingly, studies of the effects on atherosclerotic heart disease of dietary hempseed supplementation in animals or humans have not been completed. This type of study has been successfully completed using flaxseed as a dietary intervention [43,44]. It would also be important to determine if the LA content of hempseed (and not its ALA content) is responsible for decreasing inflammatory markers and the systemic atherosclerotic process in general.

### Coronary heart disease

A meta-analysis of data from 25 case-control studies strongly suggested that a lower tissue content of LA is associated with increased coronary heart disease risk [45]. More importantly, this study did not show an association between AA tissue content and the risk for coronary artery disease. The results from randomized controlled trials have not been consistent either. Some [46,47] but not all [48,49] have found reductions in coronary risk with the use of an LA diet intervention. In a recent review, Harris [45] states that reducing LA intakes to less than 5% energy would be likely to increase the risk for coronary heart disease whereas higher intakes should be beneficial even in conditions without clinical evidence of adverse effects.

## What we do not know about the effects of dietary hempseed

As discussed earlier in this paper, there is a lack of knowledge regarding the usefulness of hempseed or LA in different aspects related to cardiovascular diseases. It is important to identify not only what we presently know about dietary hempseed but also what is not known. The animal data lacks systematic information about the action of hempseed on myocardial infarctions, hypertension, atherosclerosis, markers of inflammation and arrhythmias. Similarly, we need to know more about the effects of this plant on the circulating lipid profile. Primary and secondary cardiovascular prevention trials using hempseed as a source of LA have not been performed. In general, we need to understand better the bioavailability of fatty acids like LA and GLA from dietary hempseed as a function of the age or sex of the subject, or as a function of the dosage of hempseed employed. Other dietary interventions (i.e. flaxseed) are sensitive to these variables [50,51] so it is not unrealistic to hypothesize that the delivery of hempseed will be influenced by these variables as well. It will also be important to identify if the hypotensive effects attributed to LA can be reproduced by dietary hempseed. As discussed previously, the capacity of LA and/or hempseed to affect ventricular hypertrophy secondary to high blood pressure, human atherosclerosis, inflammation, as well as the co-morbidities associated with cardiovascular diseases (like metabolic syndrome, diabetes mellitus, insulin resistance, obesity, heart failure or arrhythmias) still need to be determined in carefully controlled clinical trials.

## Conclusions

The data discussed above supports the hypothesis that hempseed has the potential to beneficially influence heart disease. A mix of legal issues and misunderstandings has slowed research progress in this area but enough data presently exists to argue strongly for the continued investigation into the therapeutic efficacy of dietary hempseed. There remain many questions regarding the cardiovascular effects of hempseed that demand scientific answers in order to definitively establish this food as a preventive or therapeutic dietary intervention. Cardiovascular patients may not be the only subjects who benefit from this research. Furthermore, only time will tell if other diseases that have an immunological, dermatological, neurodegenerative basis may also benefit from this new nutritional intervention.

## Competing interests

The authors declare that they have no competing interests.

## Authors' contributions

Both authors contributed to the creation, literature review and writing of this manuscript.
